# Supplementary material for: Interleukin-11/IL-11 Receptor Promotes Glioblastoma Cell Proliferation, Epithelial–Mesenchymal Transition, and Invasion
Source: Brain Sci. 2024 Jan 17;14(1):89. doi: 10.3390/brainsci14010089 (PMC10813507; doi:10.3390/brainsci14010089)
Supplement: Supplementary file 1 [file brainsci-14-00089-s001.zip › brainsci-2774323-supplementary.pdf]

Article

Interleukin-11/IL-11 Receptor Promotes Glioblastoma Cell Proliferation, EMT and Invasion

Sarah F. Stuart [1,2], Peter Curpen [3], Adele J. Gomes [1], Michelle C. Lan [1], Shuai Nie [4], Nicholas A. Williamson [4], George Kannourakis [,2,5], Andrew P. Morokoff [1,6], Adrian A. Achuthan [7] and Rodney B. Luwor [1,2,5*]

1 Department of Surgery, The University of Melbourne, The Royal Melbourne Hospital, Parkville, Victoria 3050, Australia.
2 Fiona Elsey Cancer Research Institute, Ballarat, Victoria 3350, Australia.
3 Townsville Hospital and Health Service, James Cook University, Townsville, Queensland 4814, Australia.
4 Melbourne Mass Spectrometry and Proteomics Facility, Bio21 Molecular Science & Biotechnology Institute, The University of Melbourne, Melbourne, VIC, Australia.
5 Federation University, Ballarat, Victoria 3350, Australia.
6 Department of Neurosurgery, The Royal Melbourne Hospital, Parkville, Victoria 3050, Australia.
7 Department of Medicine, The University of Melbourne, The Royal Melbourne Hospital, Parkville, Victoria 3050, Australia.
* Correspondence: rluwor@unimelb.edu.au

Table S1. Proteins differentially expressed in IL-11Rα transfected cells.

		20-IL-11Rα		28-IL-11Rα			
	PROTEIN NAME	FOLD CHANGE	-LOG (P-VALUE)	FOLD CHANGE	-LOG (P-VALUE)	Proliferation	Migration/Invasion
#1	RAP1B	3.34	2.94	0.69	2.72	✔	✔
#2	KYAT3	3.30	3.17	1.30	1.51		
#3	ENO3	3.11	1.96	2.17	2.73		
#4	ENO2	2.99	2.81	1.71	2.67	✔	✔
#5	WDR46	2.96	1.54	3.10	1.39	✔	
#6	NGFR	2.77	2.09	1.12	2.41		
#7	RFC1	2.70	1.64	1.48	1.58	✔	✔
#8	ALB	2.67	2.39	2.52	2.22		
#9	ASS1	2.66	2.84	2.77	5.12		
#10	ITGA7	2.44	2.48	1.02	1.78	✔	✔
#11	KYAT1	2.43	1.59	1.26	2.43		
#12	AQP1	2.23	1.46	0.79	2.48		✔
#13	FLNC	2.06	1.87	2.60	5.31		✔
#14	PTGR2	1.95	3.21	1.47	1.82		
#15	DUOX2	1.91	2.13	1.36	2.84		✔
#16	PDZRN3	1.86	1.83	2.31	2.56		✔
#17	CPPED1	1.86	1.69	2.19	1.62		
#18	MCEE	1.82	1.69	1.61	1.53		
#19	LPCAT2	1.79	2.30	3.52	2.58	✔	
#20	GALK2	1.77	2.08	1.07	2.28		
#21	LRP1	1.77	1.79	1.49	2.13		✔
#22	SPARC	1.76	2.03	1.30	2.07	✔	✔
#23	ARL6IP5	1.76	1.97	0.86	1.88		

**#24**	**AKR1B1**	1.73	1.81	0.94	2.40	✔	✔
**#25**	**SELENBP1**	1.63	2.44	3.05	1.95		
**#26**	**DNAL1**	1.60	1.77	1.20	1.77		✔
**#27**	**HSPA1L**	1.57	2.27	1.61	1.62		✔
**#28**	**CKMT1A**	1.57	1.72	1.14	1.80		
**#29**	**METTL7A**	1.53	4.74	1.20	4.41		
**#30**	**RAB5B**	1.51	2.00	1.01	1.73		✔
**#31**	**TRNT1**	1.51	1.95	0.70	2.63		
**#32**	**SARDH**	1.44	1.58	1.49	2.24		
**#33**	**NUDCD2**	1.41	1.82	1.12	4.14		✔
**#34**	**PGK1**	1.36	1.64	0.84	1.94	✔	
**#35**	**BGN**	1.35	1.75	1.45	1.90	✔	✔
**#36**	**TNC**	1.29	2.08	0.98	2.36	✔	✔
**#37**	**HSPG2**	1.22	2.02	1.29	2.86		✔
**#38**	**SELENOF**	1.17	2.63	0.92	3.11		
**#39**	**MSRB3**	1.16	1.49	1.97	2.05		
**#40**	**FAM118A**	1.15	1.65	1.33	1.58		✔
**#41**	**RSU1**	1.13	1.91	0.72	3.39		✔
**#42**	**DIABLO**	1.01	1.64	1.57	2.09		
**#43**	**ABCB11**	0.98	2.56	1.21	1.60		
**#44**	**ANXA6**	0.98	1.55	1.45	3.44		
**#45**	**NXN**	0.94	2.61	2.14	2.65	✔	
**#46**	**CKB**	0.93	3.01	1.90	3.13		✔
**#47**	**ACADL**	0.92	1.63	1.63	2.47		
**#48**	**SEPTIN9**	0.79	1.89	0.52	3.70	✔	✔
**#49**	**CYRIB**	0.77	2.69	0.48	2.82		✔
**#50**	**PLCD1**	0.76	1.81	2.30	2.00	✔	
**#51**	**UBA2**	0.69	1.85	0.53	2.99	✔	
**#52**	**ECHDC1**	0.61	2.78	1.09	2.03	✔	
**#53**	**AKR7A2**	0.53	2.80	0.61	2.65		
**#54**	**RECQL**	-0.65	2.67	-1.53	3.06		
**#55**	**TIMP3**	-0.66	1.80	-3.37	4.51		✔
**#56**	**SRP68**	-0.71	2.28	-0.66	2.68	✔	
**#57**	**AP1M1**	-0.77	2.25	-1.15	2.80	✔	
**#58**	**XRN2**	-0.86	1.98	-0.65	2.56		
**#59**	**ESYT1**	-0.87	2.82	-2.06	3.03		
**#60**	**PARP1**	-0.93	3.12	-1.60	4.04	✔	✔
**#61**	**VAC14**	-0.94	2.79	-0.68	1.93		
**#62**	**MPDU1**	-0.98	1.75	-2.80	2.55		✔
**#63**	**ELOVL5**	-1.00	1.54	-1.42	2.16	✔	✔
**#64**	**LARS1**	-1.01	2.06	-1.00	2.22	✔	✔
**#65**	**AP1G1**	-1.03	1.74	-1.08	1.77	✔	✔

**#66**	**HNRNPU**	-1.09	2.39	-0.78	2.56	✔	
**#67**	**SRP72**	-1.12	3.01	-0.72	3.18		
**#68**	**RPL15**	-1.12	1.65	-1.21	1.86	✔	
**#69**	**TNPO3**	-1.20	1.70	-0.60	2.47		✔
**#70**	**UBE2C**	-1.22	1.65	-0.88	2.05	✔	✔
**#71**	**EPS8**	-1.22	1.48	-1.12	1.67	✔	✔
**#72**	**RNPEP**	-1.41	1.63	-0.90	1.66		
**#73**	**USP10**	-1.49	2.90	-1.04	3.50	✔	
**#74**	**IARS1**	-1.49	3.10	-0.75	1.79	✔	✔
**#75**	**KIF13A**	-1.52	1.55	-0.83	2.05		✔
**#76**	**DIAPH1**	-1.53	3.44	-0.59	2.14		
**#77**	**MATR3**	-1.56	1.91	-0.95	2.15	✔	
**#78**	**RPL18A**	-2.01	2.30	-0.89	1.87	✔	
**#79**	**OSBPL10**	-2.01	2.38	-1.42	2.09		
**#80**	**CHD4**	-2.12	2.71	-1.14	2.17	✔	✔
**#81**	**ALDH1A3**	-2.19	2.13	-0.90	2.81	✔	

Note: ticks/checks for proliferation and/or migration indicate that there is published data supporting this protein in playing a role in either proliferation of migration respectively.

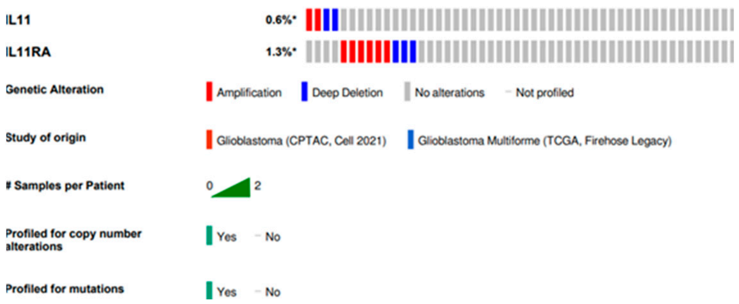

Figure S1. The Alteration Rate of IL-11 and IL-11Rα in Glioblastoma is Low. The cBioPortal was used to determine the alterations of IL-11 and IL-11Ra, including the correlative rate and alternation types.

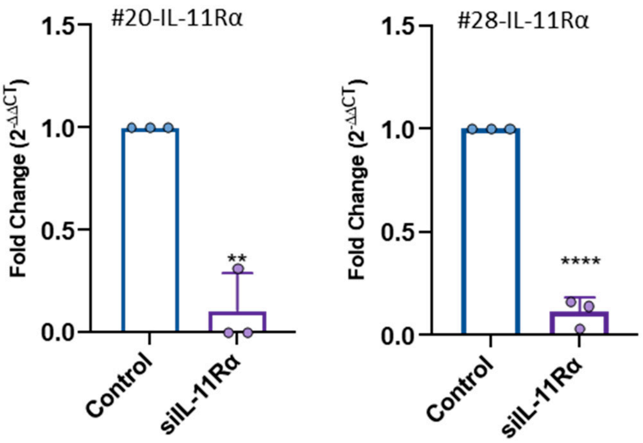

Figure S2. #20-IL-11Rα and #28-IL-11Rα cells were transfected with control or IL-11Rα siRNA and allowed to adhere overnight. qPCR was then conducted on **A.** #20-IL-11Rα and **B.** #28-IL-11Rα cells to determine the gene expression of IL-11Rα. The results have been converted to $2^{\Delta\Delta CT}$ to determine the relative fold change, in which the parental cell line was given a fold change of 1.
